# Neurofibromatosis type 1 comorbid with attention deficit and hyperactivity disorder. Case report

**DOI:** 10.1192/j.eurpsy.2022.1094

**Published:** 2022-09-01

**Authors:** P. Del Sol Calderón, R. Paricio Del Castillo, L. Mallol Castaño, A. Izquierdo De La Puente, M. Garcia Moreno

**Affiliations:** 1 Hospital Universitario Puerta de Hierro, Psiquiatría Infanto-juvenil, Madrid, Spain; 2 HOSPITAL UNIVERSITARIO PUERTA DE HIERRO MAJADAHONDA, Psychiatry, MADRID, Spain

**Keywords:** neurofibromatosis, ADHD, TICS, Comorbidity

## Abstract

**Introduction:**

A 9-year-old girl under pediatric follow-up since the age of 4 years after diagnosis of neurofibromatosis type 1

**Objectives:**

To present a case of neurofibromatosis and ADHD comorbidity to raise awareness of the importance of screening for neurodevelopmental disorders.

**Methods:**

Case report and literature review

**Results:**

The patient had an adequate control and follow-up of the disorder with periodic check-ups and magnetic resonance imaging during her follow-up. She was referred due to symptoms of inattention with failure to perform exams and impulsivity in interpersonal relationships, affecting her social functioning. In addition, the patient presented simple motor tics of eye contraction and shoulder elevation. The patient was diagnosed with attention deficit hyperactivity disorder together with tic disorder. She was treated with stimulant medication with worsening of tics and marked hyporexia. Therefore, medication with guanfacine was started up to 4 mg per day, adjusted by weight. With this dose there was a control of the tics, with improvement of the symptoms of inattention and impulsivity. In different spheres an improvement in their functionality was observed, with improvement in mood, self-esteem and academic performance.

**Conclusions:**

Neurofibromatosis type 1 is a rare monogenic disorder with a varied presentation (ophthalmologic, dermatologic and predisposition to tumor development). Patientshave been shown to present with symptoms of inattention and executive function impairment, along with other neurodevelopmental disorders such as autism spectrum disorders, learning disabilities or intellectual disability. The literature shows that up to 60% of them has ADHD criteria.

**Disclosure:**

No significant relationships.

